# Androgen Maintains Intestinal Homeostasis by Inhibiting BMP Signaling via Intestinal Stromal Cells

**DOI:** 10.1016/j.stemcr.2022.11.022

**Published:** 2023-01-10

**Authors:** Xin Yu, Shiguang Li, Yiming Xu, Yundi Zhang, Wenlong Ma, Changchun Liang, Haodong Lu, Yuge Ji, Chuanyong Liu, Dawei Chen, Jingxin Li

## Main text

(Stem Cell Reports *15*, 912–925; October 13, 2020)

In the version of this article that was initially published, there was a mistake in Figure 4C. The fifth and sixth images in the upper row of Figure 4C had overlapping fields of view. We apologize for this mistake. The fifth image in the upper row of Figure 4C has now been replaced in the corrected figure below, the other images within Figure 4C remain the same, and the interpretation of the results remains unchanged.

**Figure:**
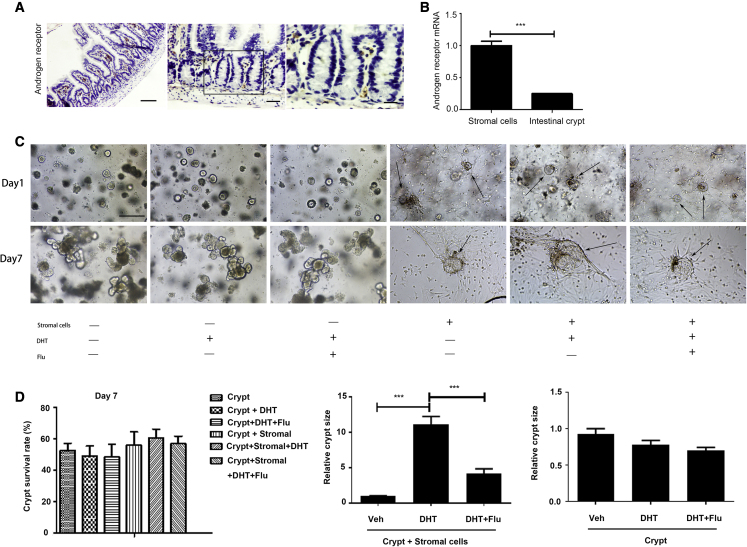
Figure 4. Androgen Promotes Organoid Expansion through Activation of AR in the Stromal Cells

