# Supplementary material for: Androgen Maintains Intestinal Homeostasis by Inhibiting BMP Signaling via Intestinal Stromal Cells
Source: Stem Cell Reports. 2020 Sep 10;15(4):912–25. doi: 10.1016/j.stemcr.2020.08.001 (PMC7561494; doi:10.1016/j.stemcr.2020.08.001)
Supplement: Document S1. Figures S1–S3 and Table S1 [file mmc1.pdf]

Stem Cell Reports, Volume *15*

Supplemental Information

Androgen Maintains Intestinal Homeostasis by Inhibiting BMP Signaling via Intestinal Stromal Cells

Xin Yu, Shiguang Li, Yiming Xu, Yundi Zhang, Wenlong Ma, Changchun Liang, Haodong Lu, Yuge Ji, Chuanyong Liu, Dawei Chen, and Jingxin Li

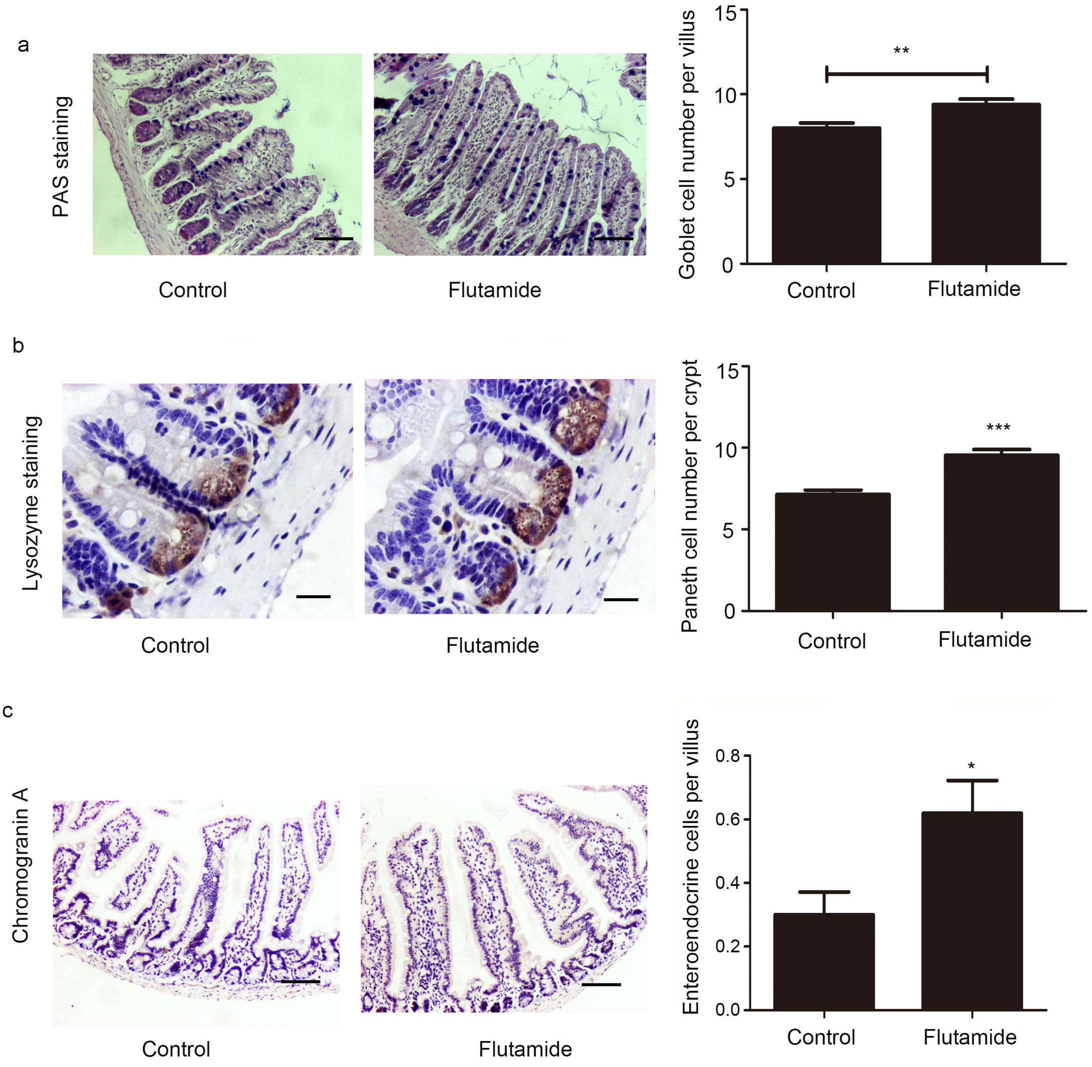

Fig.S1. Inhibition of AR model was constructed to analyze the change of three secretory lineages. Data, indicated as mean ± SD, correspond to three independent experiments (n=5 mice/group/experiment). (a) For the goblet cells count per villus in inhibition of AR model PAS staining was performed. Scale bar, 100μm. (b) For the Paneth cells count per crypt in inhibition of AR model, IHC was performed with primary antibody to lysozyme. Scale bar, 50μm, (c) For the enteroendocrine cells count per villus in the inhibition of AR model, IHC was performed with the primary antibody to chromogranin A. Scale bar, 100μm.

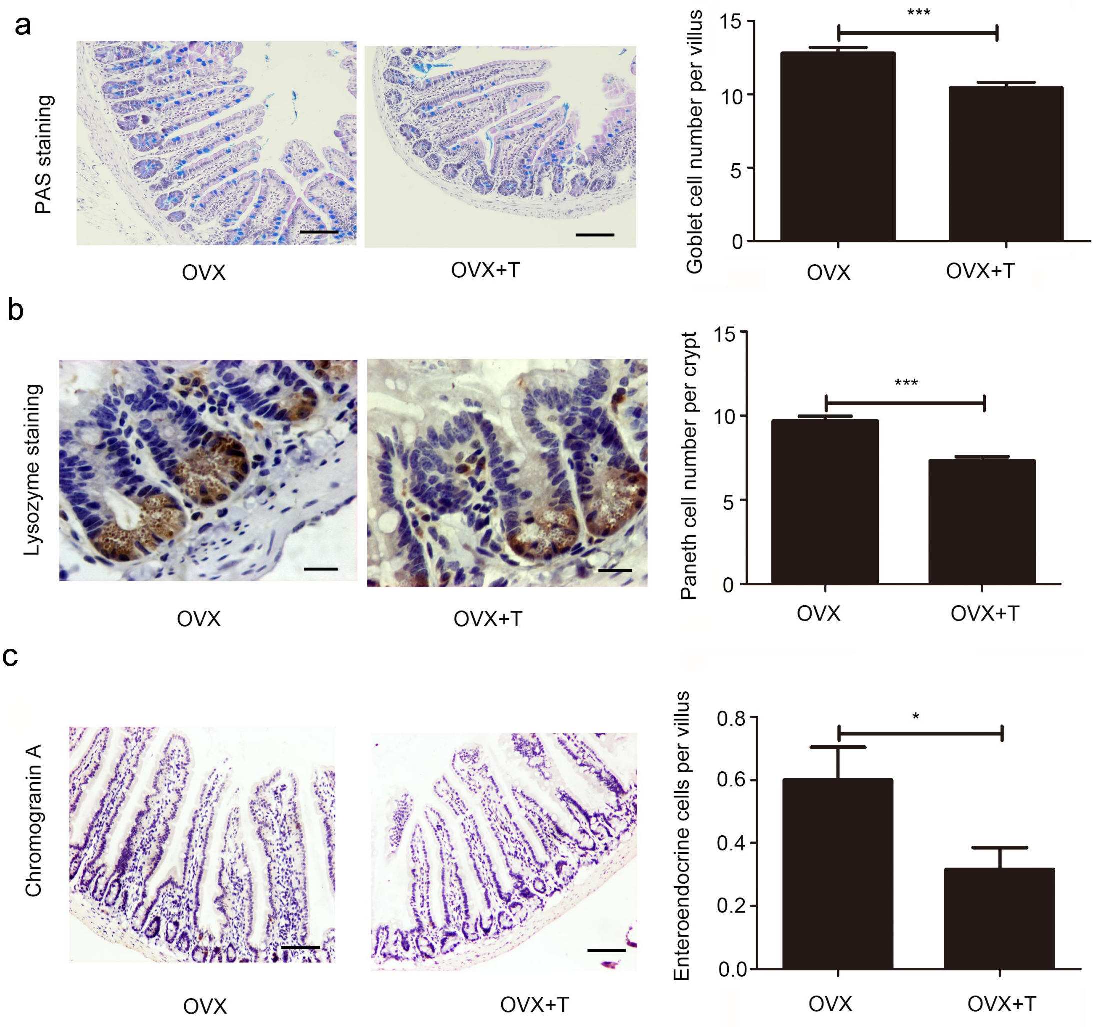

Fig.S2. OVX model was constructed to analyze the change of three secretory lineages. Data, indicated as mean ± SD, correspond to three independent experiments (n=6 mice/group/experiment). (a) For the goblet cells count per villus in OVX model, PAS staining was performed. Scale bar,100μm. (b) For the Paneth cells count per crypt in OVX model, IHC was performed with primary antibody to lysozyme. Scale bar, 50μm. (c) For the enteroendocrine cells count per villus in the OVX model, IHC was performed with the primary antibody to chromogranin A. Scale bar, 100μm.

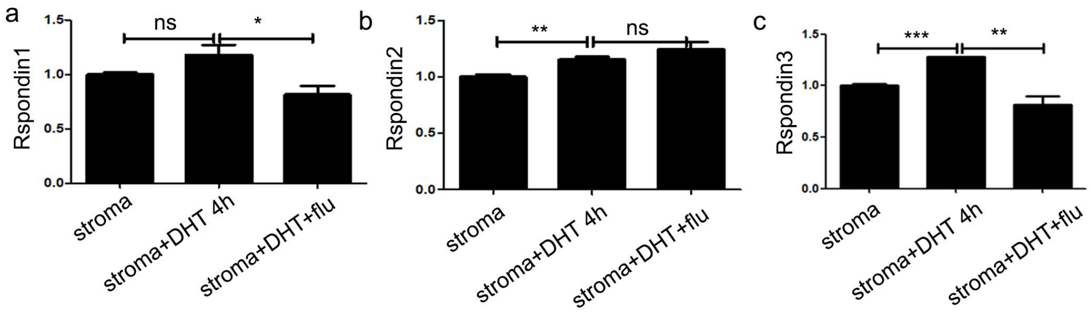

Fig.S3. Indicated mRNAs were analyzed by qRT-PCR. Data, indicated as mean ± SD, correspond to three independent experiments (n=4 mice/group/experiment). (a)*Rspo1,* (b)*Rspo2,* (c)*Rspo3.* ns means no significance. *P < 0.05, **P < 0.01, and ***P < 0.001

Table S1 Primer sequences of the analyzed genes

	forward	reverse
Ar	GGTCTTCTGGGGTGGAAAGT	GGGACCTTGGATGGAGAACT
Gadph	TGTGTCCGTCGTGGATCTGA	TTGCTGTTGAAGTCGCAGGAG
Chga	ATCCTCTCTATCCTGCGACAC	GGGCTCTGGTTCTCAAACACT
Angptl2	AAGCCATCAGGTAAGTCCTTTG	CCAGAGGATTGCCAGAGGATTA
Chrd	CCCATCCGTTCCGAGAAGG	ACTGAGGTTTGATGTTCTTGCAG
Sfrp1	CAACGTGGGCTACAAGAAGAT	GGCCAGTAGAAGCCGAAGAAC
Dkk2	CTGATGCGGGTCAAGGATTCA	CTCCCCTCCTAGAGAGGACTT
Dkk3	CTCGGGGGTATTTTGCTGTGT	TCCTCCTGAGGGTAGTTGAGA
Mmp7	CTGCCACTGTCCCAGGAAG	GGGAGAGTTTTCCAGTCATGG
Hes1	GATAGCTCCCGGCATTCCAAG	GCGCGGTATTTCCCCAACA
Wnt5a	CAACTGGCAGGACTTTCTCAA	CATCTCCGATGCCGGAACT
Wnt5b	CTGCTGACTGACGCCAACT	CCTGATACAACTGACACAGCTTT
Wnt4	AGACGTGCGAGAAACTCAAAG	GGAACTGGTATTGGCACTCCT
Wnt2b	CCGACGTGTCCCCATCTTC	GCCCCTATGTACCACCAGGA
Id3	CGACCGAGGAGCCTCTTAG	GGACGCGATAGGGAAGACC
Id2	GGTCTTCCTCCTACGAGCAG	CGATGGGCTTTAAGAGTGACAG
Msx2	TTCACCACATCCCAGCTTCTA	TTGCAGTCTTTTCGCCTTAGC
Cdkn1a	CCTGGTGATGTCCGACCTG	CCATGAGCGCATCGCAATC
Cdkn2a	CGCAGGTTCTTGGTCACTGT	TGTTCACGAAAGCCAGAGCG
Cdkn2b	CCCTGCCACCCTTACCAGA	CAGATACCTCGCAATGTCACG
Axin2	TGACTCTCCTTCCAGATCCCA	TGCCCACACTAGGCTGACA
Grem2	GGTAGCTGAAACACGGAAGAA	TCTTGCACCAGTCACTCTTGA
Nog	GCCAGCACTATCTACACATCC	GCGTCTCGTTCAGATCCTTCTC
Muc2	TTCGGCACGAGCAACTTTG	GGCAGGACACCTTGTCATTG
Bmp4	TTCCTGGTAACCGAATGCTGA	CCTGAATCTCGGCGACTTTTT

Tgfb1	CTCCCGTGGCTTCTAGTGC	GCCTTAGTTTGGACAGGATCTG
Klf4	CACCATGGACCCGGGCGTGGCTGCCAGAAA	TTAGGCTGTTCTTTTCCGGGGCCACGA
Elf3	TTCTGACTCCGGTGGAAGTGAT	CCCTTCTTATAGTCAGGAAAGCCA
Sox9	AAGTCGGTGAAGAACGGACAA	CAGCGCCTTGAAGATAGCATT
Ngn3	TCTCGCCTCTTCTGGCTTTC	CGGCAGATTTGAATGAGGGC
Atoh1	TATCCCGTCCTTCAACAACGA	TGGTCATTTTTGCAGGAAGCT
