# Supplementary material for: Androgen Maintains Intestinal Homeostasis by Inhibiting BMP Signaling via Intestinal Stromal Cells
Source: Stem Cell Reports. 2020 Sep 10;15(4):912–25. doi: 10.1016/j.stemcr.2020.08.001 (PMC7561494; doi:10.1016/j.stemcr.2020.08.001)
Supplement: Document S2. Article plus Supplemental Information [file mmc2.pdf]

Stem Cell Reports
Article
OPEN ACCESS

Androgen Maintains Intestinal Homeostasis by Inhibiting BMP Signaling via Intestinal Stromal Cells

Xin Yu,[1,5] Shiguang Li,[4,5] Yiming Xu,[2,5] Yundi Zhang,[1] Wenlong Ma,[1] Changchun Liang,[1] Haodong Lu,[1] Yuge Ji,[1] Chuanyong Liu,[1] Dawei Chen,[3,*] and Jingxin Li[1,*]
[1]Department of Physiology, School of Basic Medical Sciences, Cheeloo College of Medicine, Shandong University, 250012 Jinan, China
[2]Department of Orthopaedics, Peking Union Medical College Hospital, Chinese Academy of Medical Sciences & Peking Union Medical College, No. 1 Shuaifuyuan Wangfujing Dongcheng District, 100730 Beijing, China
[3]Laboratory of Medical Chemistry, Interdisciplinary Cluster for Applied Genoproteomics (GIGA), University of Liège, CHU, Sart-Tilman, 4000 Liège, Belgium
[4]School of Clinical Medicine, Cheeloo College of Medicine, Shandong University, 250012 Jinan, China
[5]Co-first author
*Correspondence: dawei.chen@uliege.be (D.C.), ljingxin@sdu.edu.cn (J.L.)
https://doi.org/10.1016/j.stemcr.2020.08.001

SUMMARY

Research shows a higher incidence of colorectal cancer in men. However, the molecular mechanisms for this gender disparity remain unknown. We report the roles of androgen in proliferation and differentiation of intestinal stem cells via targeting of the androgen receptor (AR) on intestinal stromal cells by negatively regulating BMP signaling. Orchidectomy (ORX) or the AR antagonist promotes expansion of intestinal epithelium but suppresses intestinal stem cell (ISC) proliferation. Conversely, the AR agonist inhibits ISC differentiation but augments proliferation in ovariectomized mice. Mechanistically, activation of the AR increases expression of BMP antagonists but lowers expression of BMP4 and Wnt antagonists in primary stromal cells, which promotes intestinal organoid growth. Interestingly, the BMP pathway inhibitor LDN-193189 reverses the ORX-induced effects. Our results highlight that stromal cells constitute the intestinal stem cell niche and provide a possible explanation for higher incidence rates of colorectal cancer in men.

INTRODUCTION

The intestinal epithelium structure is organized into villi and crypts and rapidly self-renews every 5 days (Barker, 2014; Clevers, 2013). Intestinal stem cells (ISCs) localized at the base of the crypts produce progenitors. These progenitors proliferate and differentiate into six separate mature epithelial cell types, comprising enterocytes, M cells, Paneth cells, goblet cells, enteroendocrine cells, and tuft cells (van der Flier and Clevers, 2009). Niches are specialized and conducive microenvironments in which stem cells reside. Niches provide all of the essential factors for ISC renewal, growth, and function (Barker, 2014). In addition, intestinal epithelial cells and mesenchymal cells, which are believed to be the principal source of canonical Wnt signaling and an exclusive source of noncanonical Wnt signaling, and Wnt antagonists, bone morphogenetic proteins (BMPs) and BMP antagonists, are also important components of the ISC niche (Powell et al., 2011; Shoshkes-Carmel et al., 2018). Colorectal cancer is one of the most frequently diagnosed cancers in the world. Significant sex differences are observed in colon cancer, with a higher ratio of colorectal cancer occurring in men (Ferlitsch et al., 2011). Ovariectomy (OVX) in female rats did not show any effect on the prevalence of adenomas, while orchidectomy (ORX) in male rats distinctly prevented tumor development (Amos-Landgraf et al., 2014). Men have a higher risk of developing colon cancer and a lower survival rate than women. Sex differences in colon cancer have been largely attributed to sex hormones, but the molecular features that drive these sex differences remain poorly understood. It was previously shown that the constitution and stem cell microenvironment between intestinal and colonic epithelia are similar (Horvay and Abud, 2013). In this study, we report that the androgen receptor (AR) is expressed in the intestinal mesenchymal cells but not in the intestinal epithelial cells. *In vitro* experiments have proven that activation of AR in response to dihydrotestosterone (DHT) leads to higher expression of BMP antagonists while reducing expression of both BMP4 and Wnt antagonists in cultured primary stromal cells, which promotes the expansion of organoids. In addition, the numbers of intestinal secretory epithelial lineages and enterocytes increased, while the numbers of S-phase cells were reduced 2 weeks after ORX or administration of AR antagonist in male mice. Our data highlight a previously unappreciated role for androgens in the ISC niche, supporting ISC proliferation and restraining epithelium differentiation by negatively regulating BMP signaling in the stromal cells. These findings may help explain the observed higher incidence rates of colorectal cancer in men.

RESULTS

Androgens Decrease the Population of Differentiated Cells in the Intestinal Epithelium

ORX, OVX, and inhibition of AR in male mouse models were done to investigate the effect of androgens on ISC terminal

This is an open access article under the CC BY license (http://creativecommons.org/licenses/by/4.0/).

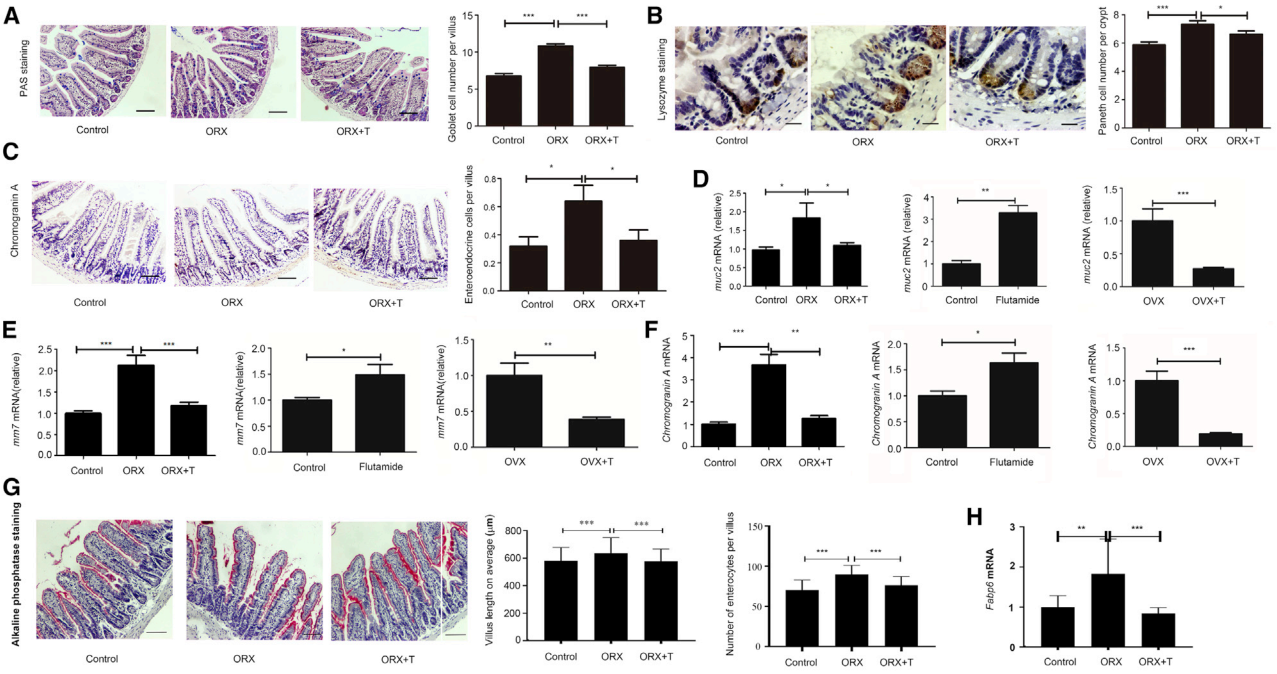

Figure 1. Androgen's Effect on ISC Lineage Specification

ORX, OVX, and inhibition of AR models were constructed to analyze the change in three secretory lineages and enterocytes. For counting of differentiated cells, 10 villi were randomly chosen for the goblet cells, enteroendocrine cells, and enterocytes, and 10 crypts were randomly captured for the Paneth cells. Ilea were isolated from all mice for immunohistochemistry (IHC), RNA isolation, and quantitative real-time PCR. Data, indicated as mean ± SD, correspond to three independent experiments (A–C, G, n = 6 mice/group/experiment; D–F, H, n = 4 mice/group/experiment).

(A) For the goblet cell count per villus in the ORX model, PAS staining was performed. n = 6. Scale bar, 100 μm.

(B) For the Paneth cell count per crypt in the ORX model, IHC was performed with primary antibody to lysozyme. Scale bar, 50 μm.

(C) For the enteroendocrine cell count per villus in the ORX model, IHC was performed with the primary antibody to chromogranin A. Scale bar, 100 μm.

(D) For determination of relative mRNA levels of *muc2* in all groups, quantitative real-time PCR was done.

(E) For determination of relative mRNA levels of *mmp7* in all groups, quantitative real-time PCR was done.

(F) For determination of relative mRNA levels of *Chromogranin A* in all groups, quantitative real-time PCR was done.

(G) For the villus length and enterocyte count per villus in the ORX model, alkaline phosphatase staining was performed. Scale bar, 100 μm.

(H) For determination of relative mRNA levels of *Fabp6* in all groups, quantitative real-time PCR was done. ORX+T, supplemented with DHT after ORX.

$^{*}p < 0.05$, $^{**}p < 0.01$, and $^{***}p < 0.001$.

differentiation. The small intestines were isolated for specific staining and quantification of secretory cells and enterocytes. Histological analysis was performed on the three chief secretory cell types and enterocytes: periodic acid-Schiff (PAS) to stain goblet cells, anti-chromogranin A to stain enteroendocrine cells, anti-lysozyme to stain Paneth cells, and alkaline phosphatase to stain enterocytes (van der Flier and Clevers, 2009). Our results indicated that the goblet cells, enteroendocrine cells, and Paneth cells were all increased in males after ORX compared with controls, and this phenomenon was reversed by supplementation with DHT (Figures 1A–1C). Investigation of enterocytes was consistently similar (Figure 1G). Treatment with 2-hydroxy flutamide increased the secretory cells in the intestine (Figures S1A–S1C). In addition, administration of DHT decreased the numbers of all three secretory lineages in OVX females (Figures S2A–S2C). In summary, androgens may decrease the numbers of secretory lineages and enterocytes. Specific molecules expressed by a certain secretory cell type were regarded as biomarkers. Therefore, mRNA levels of these biomarkers were detected to observe the changes in secretory lineages. Quantitative real-time PCR was performed to analyze the expression of cellular markers for these secretory lineages: mucin2 (*Muc2*) for goblet cells, chromogranin A for enteroendocrine cells, matrix metalloproteinase7 (*Mmp7*) for Paneth cells, and fatty acid binding protein6 (*Fabp6*) for enterocytes (Haber et al., 2017; Porter et al., 2002). All three markers for these secretory lineages and enterocytes were upregulated in ORX males,

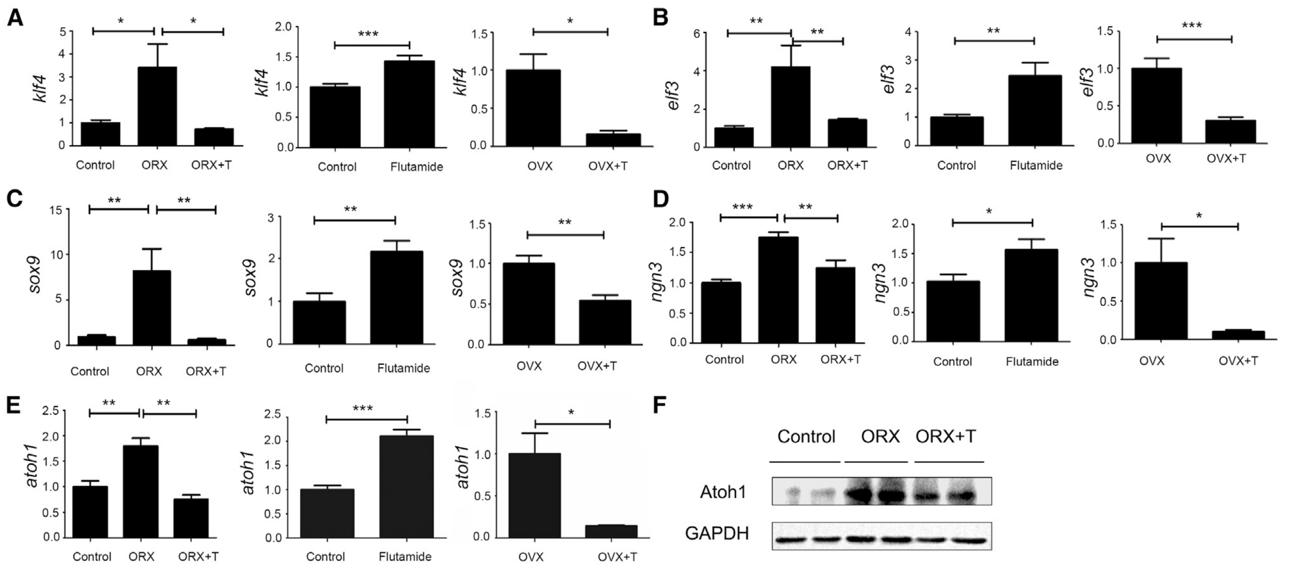

Figure 2. The Mechanism Underlying Androgen's Effect on ISC Differentiation

Quantitative real-time PCR was performed to investigate the mRNA levels of differentiation-related factors. Data, indicated as mean ± SD, correspond to three independent experiments (n = 4 mice/group/experiment).

(A and B) For the goblet cell fate specification, the relative mRNA levels of (A) Klf4 and (B) Elf3 were detected in the ORX, OVX, and inhibition of AR models.

(C) For the Paneth cell fate specification, the relative mRNA levels of Sox9 were detected in the ORX, OVX, and inhibition of AR models.

(D) For the enteroendocrine cell fate specification, the relative mRNA levels of Ngn3 were detected in the ORX, OVX, and inhibition of AR models.

(E) For the secretory lineage specification, the relative mRNA levels of atoh1 were detected in the ORX, OVX, and inhibition of AR models.

(F) The expression of Atoh1 was assessed by western blot analysis.

$^{*}p < 0.05$, $^{**}p < 0.01$, and $^{***}p < 0.001$

and the addition of exogenous androgens reversed this phenomenon. Males treated with 2-hydroxy flutamide expressed higher levels of these markers than controls. OVX females injected with DHT expressed lower mRNA levels of the markers mentioned above than untreated OVX mice (Figures 1D–1F and 1H).

Androgens Regulate the Expression of Differentiation-Related Factors

Wnt and Notch signaling pathways are vital to the proliferation and differentiation of ISCs. Downstream target signals include atonal homolog 1 (*Atoh1*), Sry-related HMG box 9 (*Sox9*), Krüppel-like factor 4 (*Klf4*), E47-like factor 3 (*Elf3*) and Neurogenin3 (*Ngn3*) (Ghaleb et al., 2011; Jenny et al., 2002; Mori-Akiyama et al., 2007; van der Flier and Clevers, 2009; Vanuytsel et al., 2013). Quantitative real-time PCR was performed to assess downstream molecules of both signaling pathways to explore the mechanism underlying androgens' effects on cell lineage specification in our mouse model. Sox9 directly drives the differentiation into Paneth cells, *Ngn3* for the enteroendocrine cells, and *Klf4* and *Elf3* for the goblet cells. Expression of *Sox9*, *Ngn3*, *Klf4*, and *Elf3* was upregulated in the ORX males and supplementation with DHT reversed these effects. Treatment with 2-hydroxy flutamide promoted the expression of *Sox9*, *Ngn3*, *Klf4*, and *Elf3* at the mRNA level. OVX females injected with DHT exhibited lower expression levels than their respective controls (Figures 2A–2D). *Atoh1* is a common upstream gene for *Sox9*, *Ngn3*, *Klf4*, and *Elf3*. *Atoh1* expression was promoted in males depleted of androgens by ORX and this observation was reversed by the addition of DHT (Figure 2F). Treatment with 2-hydroxy flutamide caused higher expression levels of *Atoh1* at the mRNA level. OVX females injected with DHT expressed reduced *Atoh1* levels compared with controls (Figure 2E). Taken together, these findings indicate that androgens might inhibit the expression of secretory lineage-related differentiation-determined factors.

Androgens Promote ISC Proliferation through Upregulation of Active β-catenin and Downregulation of Bmp4

We next aimed to establish changes in the proliferation status of ISCs after studies demonstrated an increase in differentiated epithelial cells. All the mechanisms underlying our observations were investigated through manipulation of hormonal status as previously mentioned.

Bromodeoxyuridine (BrdU) was utilized as a label to determine whether androgens improve the proliferation of stem cells (Kyryachenko et al., 2012). Males with ORX exhibited fewer $BrdU^+$ cells than the controls, while supplementation with DHT seemed to reverse this phenomenon (Figure 3A). Treatment with 2-hydroxy flutamide decreased the number of $BrdU^+$ cells in the males (Figure 3B). And injection with DHT increased the number of $BrdU^+$ cells in the OVX females (Figure 3C). In conclusion, androgens are likely to be involved in the enlargement of S-phase cells in the intestinal epithelia. The Wnt pathway drives the proliferation, while the BMP pathway inhibits the proliferation (van der Flier and Clevers, 2009). Western blotting was conducted to analyze the expression of β-catenin, the central player in the Wnt pathway, which contributes to the proliferation of intestinal epithelial stem cells. Expression of both active β-catenin (nonphosphorylated β-catenin) and total β-catenin was investigated. Western blot analysis demonstrated that the depletion of androgens by ORX downregulated active β-catenin, while ORX males administered DHT presented no significant differences from the controls (Figure 3D). We also used Olfm4 to identify the ISCs by immunohistochemistry (IHC) (van der Flier et al., 2009). There were fewer $OLfm4^+$ stem cells in the males with ORX, but the number of $Olfm4^+$ cells increased to normal levels after supplementation with DHT (Figure 3E). The BMP signaling pathway was investigated by quantitative real-time PCR because BMPs restrict the stemness of ISCs by inhibiting gene expression (Qi et al., 2017). Expression of *Bmp4* and BMP type I receptor A (*Bmpr1a*) was upregulated in ORX males compared with controls. Supplementation with DHT inhibited the mRNA levels of *Bmp4*. Expression of *Bmp4* was suppressed by administration of exogenous androgens in OVX females (Figure 3F). Immunoblotting of Smad1/5 activity confirmed that the BMP pathway contributes to the effect of androgens on intestinal epithelium in the ORX models (Figure 3G). Consequently, androgens might promote proliferation by improving Wnt pathway signaling and restraining the BMP pathway.

Activation of AR in the Stromal Cells Promotes Intestinal Organoid Expansion *In Vitro*

IHC results of the small intestine from wild-type C57BL/6J mice indicated that no AR-positive cells were present in the intestinal epithelium, while a number of stromal cells expressed AR (Figure 4A). We analyzed the levels of AR expression in intestinal epithelial cells through quantitative real-time PCR (Figure 4B). Statistical results also showed that AR was more highly expressed in stromal cells than in crypts (Figure 4B), implying that DHT may act on stromal cells. We next cocultured stromal cells and intestinal epithelial crypts *in vitro* to further investigate the target cells of DHT. There was a significant difference between individually cultured crypts and cocultured cells. A comparison between crypts cocultured with stromal cells (crypt + stroma) and cocultured plus DHT (crypt + stroma + DHT) on day 7 demonstrated that the sizes of crypts cocultured with stromal cells were increased significantly in response to the addition of DHT, while addition of the AR antagonist flutamide counteracted the effects of DHT on crypt size (Figure 4C). The statistical results of crypt size on day 7 also demonstrated that DHT had no effect on proliferation of crypts cultured alone. However, DHT significantly promoted the proliferation of crypts in the cocultured group (Figure 4D). There was no difference in survival rates of crypts among the four groups (Figure 4D). This phenomenon suggests that DHT may act on stromal cells to enhance the proliferation of crypts.

Androgen Regulated BMP-Related Gene Expression in Intestinal Stromal Cells

The results of coculture experiments suggested that DHT influences the proliferation of crypts primarily through stromal cells. The expression of Wnt-related signals produced by stromal cells was next examined to determine whether DHT promotes intestinal epithelial crypt proliferation through Wnt pathways. Cells were treated with DHT for 4 h, but no significant changes were observed in *wnt4*, *wnt5a*, *wnt5b*, or *wnt2b* (Figure 5A). R-spondins are critical molecules that promote ISC division. Therefore, we also assessed the expression of *Rspo1*, *Rspo2*, and *Rspo3* (Sigal et al., 2017; Yan et al., 2017), which still revealed no significant differences between control and DHT-treated groups. We subsequently examined the expression of Wnt-related antagonists and showed that the expression of *Dkk2*, *Dkk3*, and *Sfrp1* was significantly reduced after treatment with DHT (Figure 5B). We next detected changes in the BMP/transforming growth factor β (TGF-β) pathway as BMP signaling is also involved in ISC self-renewal. It turned out that *Bmp4* and *Tgfb1* were downregulated (Figure 5C). We further investigated the expression of BMP-related antagonists, and observed a significant increase in BMP signaling antagonism (Figure 5D). In addition, flutamide treatment inhibited the function of DHT in the tested genes (Figures 5B–5D). We primarily observed decreases in *Cdkn1a*, *Cdkn2a*, and *cdkn2b*, downstream genes of the TGF-β signaling pathway (Figure 5E), and BMP target genes *Id2*, *Id3*, and *Msx2* (Figure 5F). Therefore, it was elucidated that DHT exaggerated the expression of BMP antagonists to inhibit the BMP signaling pathway, which subsequently affected the expansion of the crypt population *in vitro* (Auclair et al., 2007; Gregorieff et al., 2005; Tian et al., 2017).

Selective Inhibition of the BMP Pathway Mimics Androgen Effects

Given our results, we next focused on the BMP pathway. Inhibition of the BMP pathway model was performed to

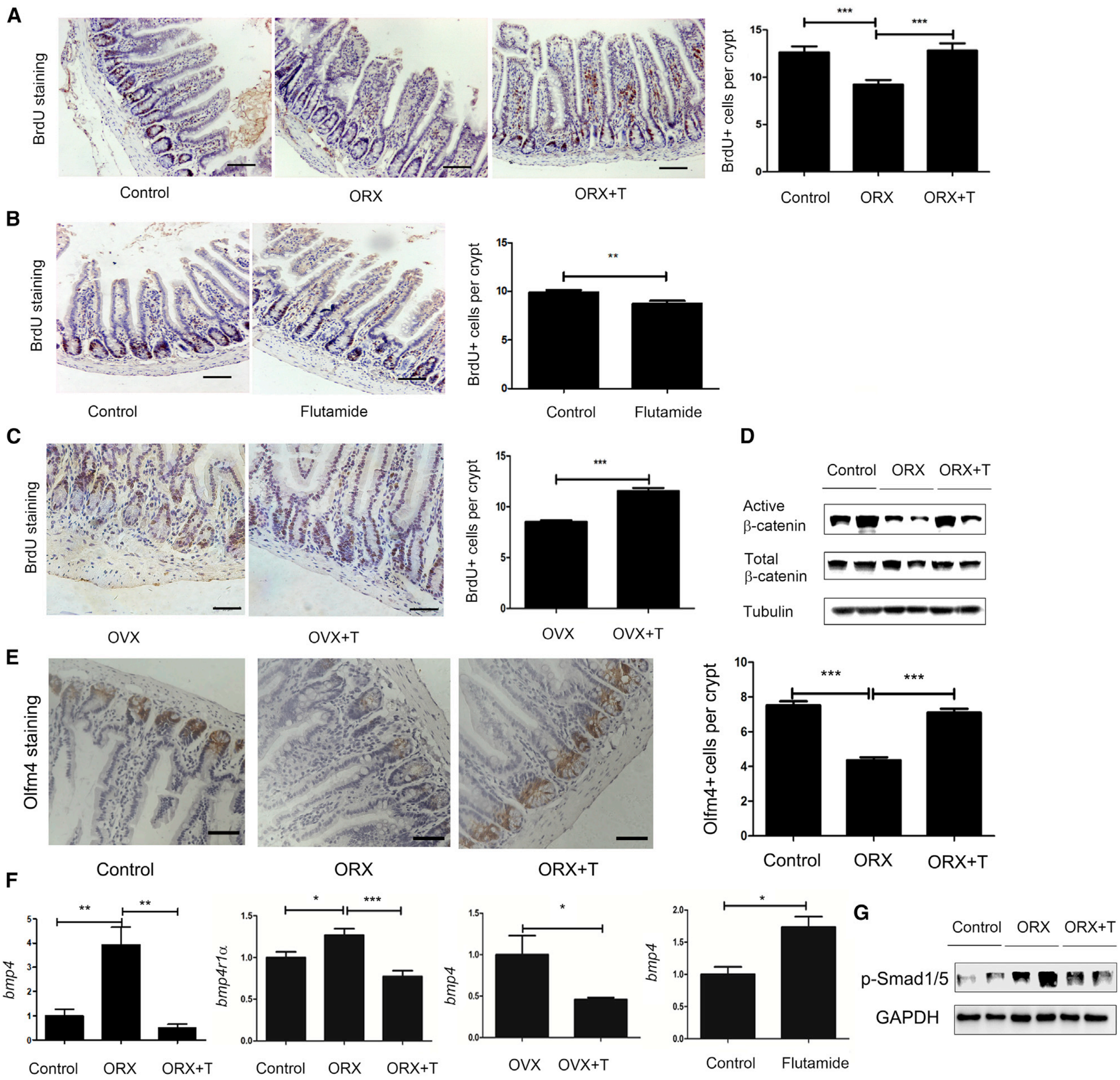

Figure 3. The Effect of Androgen on ISC Proliferation

For counting of BrdU+ cells, 10 crypts were randomly chosen for investigation.

(A) For detection of BrdU+ cells per crypt in the ORX model, IHC was performed with primary antibody to BrdU. Scale bar, 100 μm.

(B) For detection of BrdU+ cells per crypt in the inhibition of AR model, IHC was performed with primary antibody to BrdU. Scale bar, 100 μm.

(C) For detection of BrdU+ cells per crypt in the OVX model, IHC was performed with primary antibody to BrdU. Scale bar, 100 μm.

(D) For analysis of active β-CATENIN and total β-CATENIN expression, western blotting was performed.

(E) For detection of intestinal stem cells in the ORX model, IHC was performed with primary antibody to Olfm4+. Scale bar, 50 μm.

(F) For analysis of relative mRNA levels of *Bmp4*, quantitative real-time PCR was performed in the ORX, OVX, and inhibition of AR models.

(G) The phosphorylation of Smad1/5 was assessed by western blot analysis.

Data, indicated as mean ± SD, correspond to three independent experiments (A, C, n = 6; B, n = 5; D, G, n = 2; E, F, n = 4 mice/group/experiment). $^*p < 0.05$, $^{**}p < 0.01$, and $^{***}p < 0.001$.

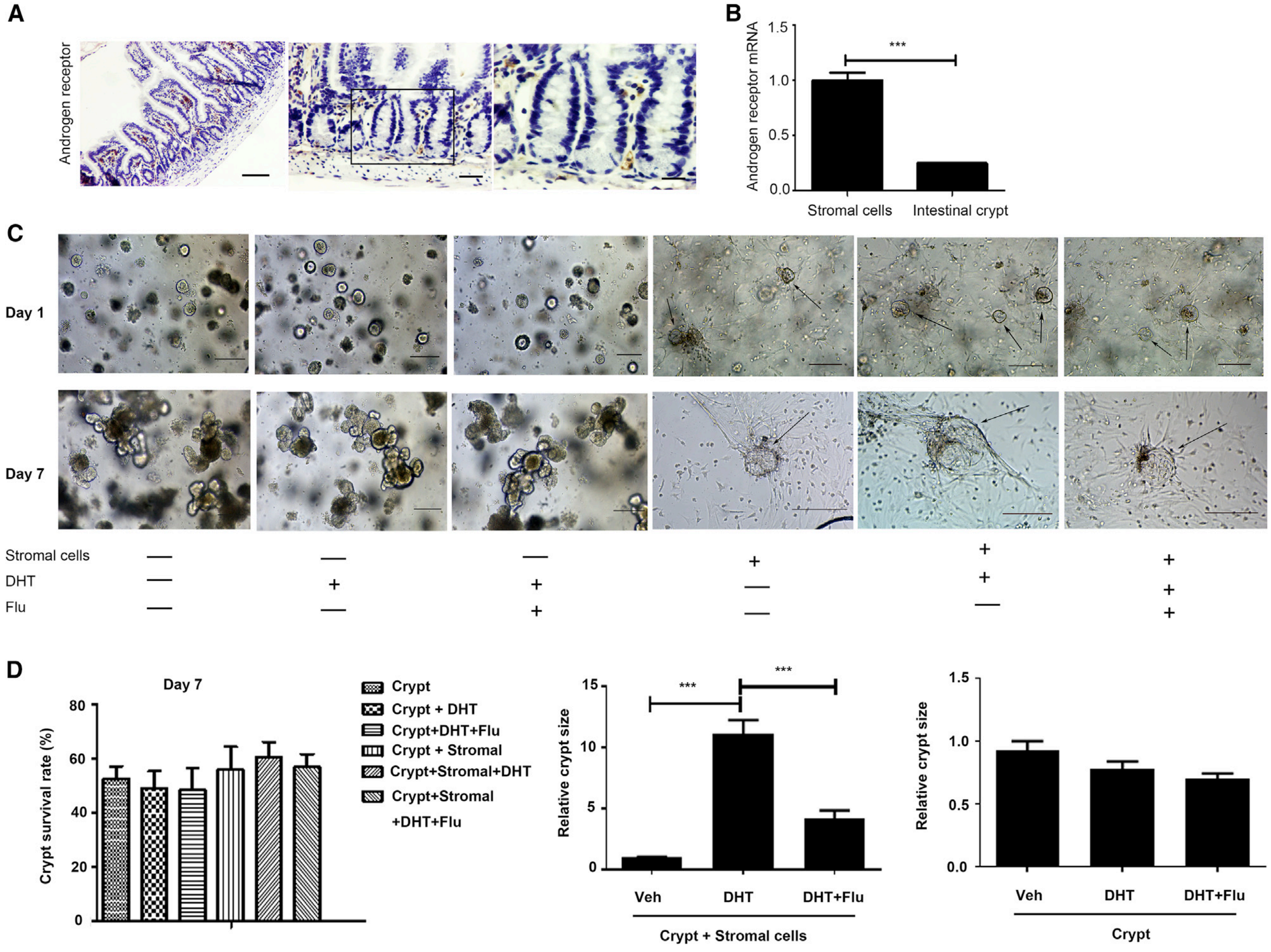

Figure 4. Androgen Promotes Organoid Expansion through Activation of AR in the Stromal Cells

(A) AR immunostaining on small intestine epithelium isolated from wild-type mice. Left, original magnification ×10. Scale bar, 100 μm. Middle, original magnification ×20. Scale bar, 50 μm. Right, original magnification ×40. Scale bar, 20 μm.

(B) mRNA levels determined by quantitative real-time PCR in intestinal epithelium.

(C) Wild-type organoids in complete medium, organoids plus DHT (1×10^{-7}mol/L), and organoids cocultured with stromal cells plus DHT (1×10^{-7}mol/L) and flutamide (Flu; 2 μM). Comparison of cocultured cell size about day 1 and day 7 is shown. All the pictures of cultured crypts were ×10 original magnification (scale bar, 100 μm); black arrows, crypt spheroid.

(D) Survival rate graph obtained by counting the number of crypts on day 7 (left). Statistics on the size of the 7 days cocultured (crypt + stromal cells) and cocultured plus DHT (middle); crypt and crypt plus DHT (right)

Data, indicated as mean ± SD, correspond to three independent experiments. ***$p < 0.001$.

investigate the mechanism underlying the regulation of male hormones upon proliferation and differentiation. First, quantitative real-time PCR was performed to analyze downstream molecules of the BMP pathway: msh homeobox 1 (*Msx1*) and inhibitors of DNA binding (*Id1*) (Tian et al., 2017). Figure 6A shows that both factors were enhanced by ORX compared with controls. Use of LDN193189, a BMP pathway inhibitor, reversed the effect caused by ORX. Therefore, LDN193189 was proven to be valid. Immunohistochemical analysis was subsequently performed for the goblet cells, Paneth cells, enteroendocrine cells, BrdU$^+$ cells, and Olfm4$^+$ cells. The results indicated that an increase in all three secretory lineages caused by ORX and decrease in BrdU$^+$ cells and Olfm4$^+$ cells caused by ORX were reversed by the addition of LDN193189 (Figures 6B–6E). Thus, BMP pathway inhibition and androgens may exert similar effects on ISC proliferation and differentiation in ORX mice, as illustrated in Figures 1 and 2.

DISCUSSION

The incidences of several cancer types arising in organs with nonreproductive functions are significantly higher in males than in females, with associated differences in

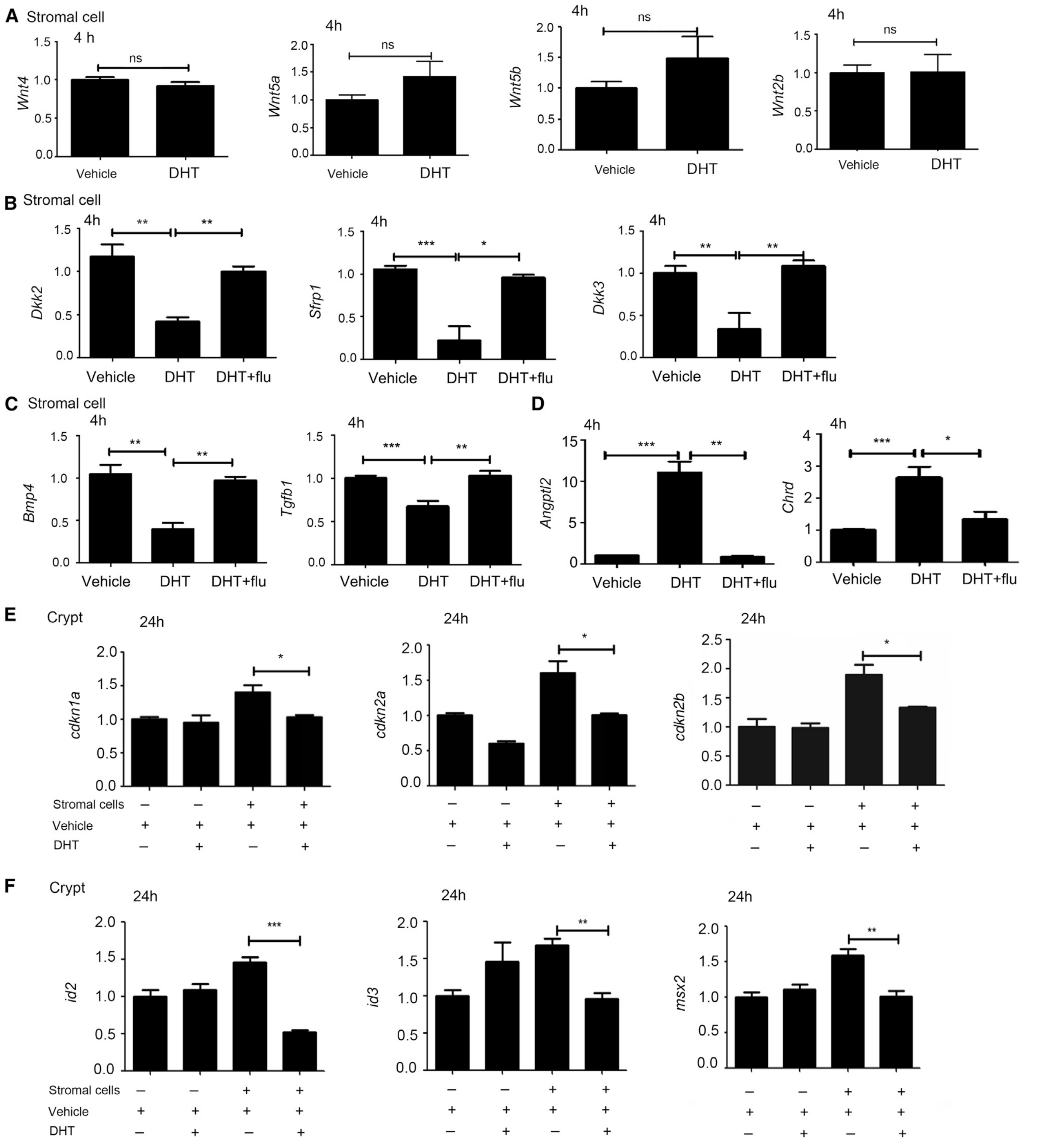

Figure 5. Androgen Regulated the BMP-Related Gene Expression in Intestinal Stromal Cells

(A) Indicated mRNAs were analyzed by quantitative real-time PCR. Values were normalized to *Gapdh* expression and expressed relative to each gene of the Wnt pathway.

(B) Quantitation of antagonists of the Wnt signal pathway.

(C and D) mRNA levels determined by quantitative real-time PCR of (C) *Tgfb1* and *Bmp4* and (D) antagonists of BMP. In (A), (B), (C), and (D), all the cDNAs used were synthesized from stromal cells, stromal cells with DHT, and stromal cells with DHT and flutamide for 4 h.

(legend continued on next page)

survival. Sex differences are observed during the development and progression of various types of cancers arising in organs without reproductive functions, such as colon (Ferlitsch et al., 2011), rectum, liver, esophagus and stomach, larynx, lung and bronchus, and skin, and males have higher risks and higher mortality rates for these cancers than females (Siegel et al., 2015; Torre et al., 2015). Recent colorectal cancer statistics showed a much higher incidence of colorectal cancer in men (48.1 per 100,000 population) than in women (38.2 per 100,000 population) (Siegel et al., 2017). Even though the higher risk in males might be partially attributed to occupational exposures and/or behavioral factors such as diet, smoking, and alcohol consumption, males still exhibit a higher cancer risk after adjusting for these risk factors (Clocchiatti et al., 2016; Edgren et al., 2012; Wei et al., 2004). Colorectal cancer is one of the most prevalent malignant diseases worldwide. However, the signaling pathways and driven genes involved are largely unclear. Understanding sex differences in colorectal cancer is essential in advancing disease prevention, diagnosis, and treatment. James et al. (Amos-Landgraf et al., 2014) reported that depletion of endogenous male hormones by ORX might prevent adenomagenesis in an AOM mouse model of adenomagenesis, suggesting that male hormones may be responsible for colon carcinogenesis. However, a concrete molecular mechanism underlying the effect of androgens on colonic epithelial proliferation and differentiation, as well as intestinal epithelial proliferation and differentiation, remains unclear. Our study aimed to establish the underlying role of male hormone androgens in ISCs from a new perspective.

BMPs belong to the TGF-β superfamily of ligands, and BMP signaling constrains ISC expansion and boosts epithelial differentiation by antagonizing Wnt signaling in the crypts (Sneddon et al., 2006). Smad1 is phosphorylated and activated by BMPs in androgen-sensitive prostate cancer, and it interacts with AR to suppress its function (Qiu et al., 2007). The consensus view of prostate cells is that androgens bind to AR elements to inhibit TGF-β expression. TGF-β has been detected in the prostatic stromal cells but not in the epithelial cells as a potent epithelial cell-inhibiting agent (Lee, 1996). These findings suggest that androgens might also stimulate intestinal stromal cells to generate TGF-β to suppress the proliferation of intestinal epithelial cells. The intestine was selected due to its strong active renewal ability and various types of differentiated epithelial cells. Androgens that combine with AR to produce downstream signaling are considered effective androgens. The ORX, OVX, and inhibition of AR models are built through operation or injection of a competitive inhibitor of AR to control the levels of effective androgens, in which 2-hydroxy flutamide, an AR antagonist, functions as chemical castration. Staining and quantitative analysis indicated that androgens decrease the number of Paneth cells, goblet cells, enteroendocrine cells, and enterocytes, preventing ISCs from specifying into well-differentiated epithelial cells. In addition, this study proved that differentiation-related factors (*Sox9*, *Ngn3*, *Klf4*, and *Elf3*) were downregulated in response to androgen, which is consistent with histological observation.

Several mechanisms influence ISC renewal, including the Wnt/β-catenin pathway, which promotes proliferation, and the BMP pathway, which inhibits proliferation. β-catenin is the central player in the canonical Wnt pathway and mediates signaling that is responsible for proliferation. Reduction of intestinal BrdU^{+} cells by endogenous androgen depends on a reduction of active β-catenin. BMP suppresses proliferation through inhibition of the Wnt pathway (He et al., 2004). Androgens downregulate the expression of *Bmp4*, subsequently weakening BMP's inhibitory effect and facilitating Wnt's promotion. Overall, androgens stimulate proliferation.

We subsequently identified the location and expression of AR in the small intestine. IHC results exhibited that low levels of AR occur in intestinal stromal cells, which is consistent with the finding of James et al. (Amos-Landgraf et al., 2014). Quantitative analysis elucidates that expression of AR mRNA in stromal cells is several times higher than in crypts. Studies imply that the target cells of androgens are a stromal cell population in the intestinal lamina propria that is capable of inducing the production of BMP antagonists. The crypt-stroma coculture model demonstrated a stromal-dependent promotion of ISC proliferation by androgens. DHT has no effect on isolated crypts, and expansion of the crypt population was observed only when stroma was cocultured with crypts, which appears as an enlargement of crypt size when viewed microscopically. However, we cannot dismiss the possibility that there are other indirect effects on the ISCs induced by androgens *in vivo*, which requires further investigation.

Mesenchyme is an essential component of the ISC niche. Wnt-related signals and BMP-related signals that are secreted and mediated by the mesenchyme are significant in ISC proliferation and differentiation (Auclair et al., 2007; Gregorieff et al., 2005). Three Wnt antagonists (*Sfrp1*, *Dkk2*, and *Dkk3*) are downregulated *in vitro* by androgens, indirectly inducing upregulation of the

(E and F) Quantitation of (E) *Cdkn1a*, *Cdkn2a*, and *Cdkn2b* (TGF-β downstream genes) and (F) *Id2*, *Id3*, and *Msx2* (BMP downstream genes). In (E) and (F), values are means + SEM. Data, indicated as mean ± SD, correspond to three independent experiments.
$^{*}p < 0.05$, $^{**}p < 0.01$, and $^{***}p < 0.001$.

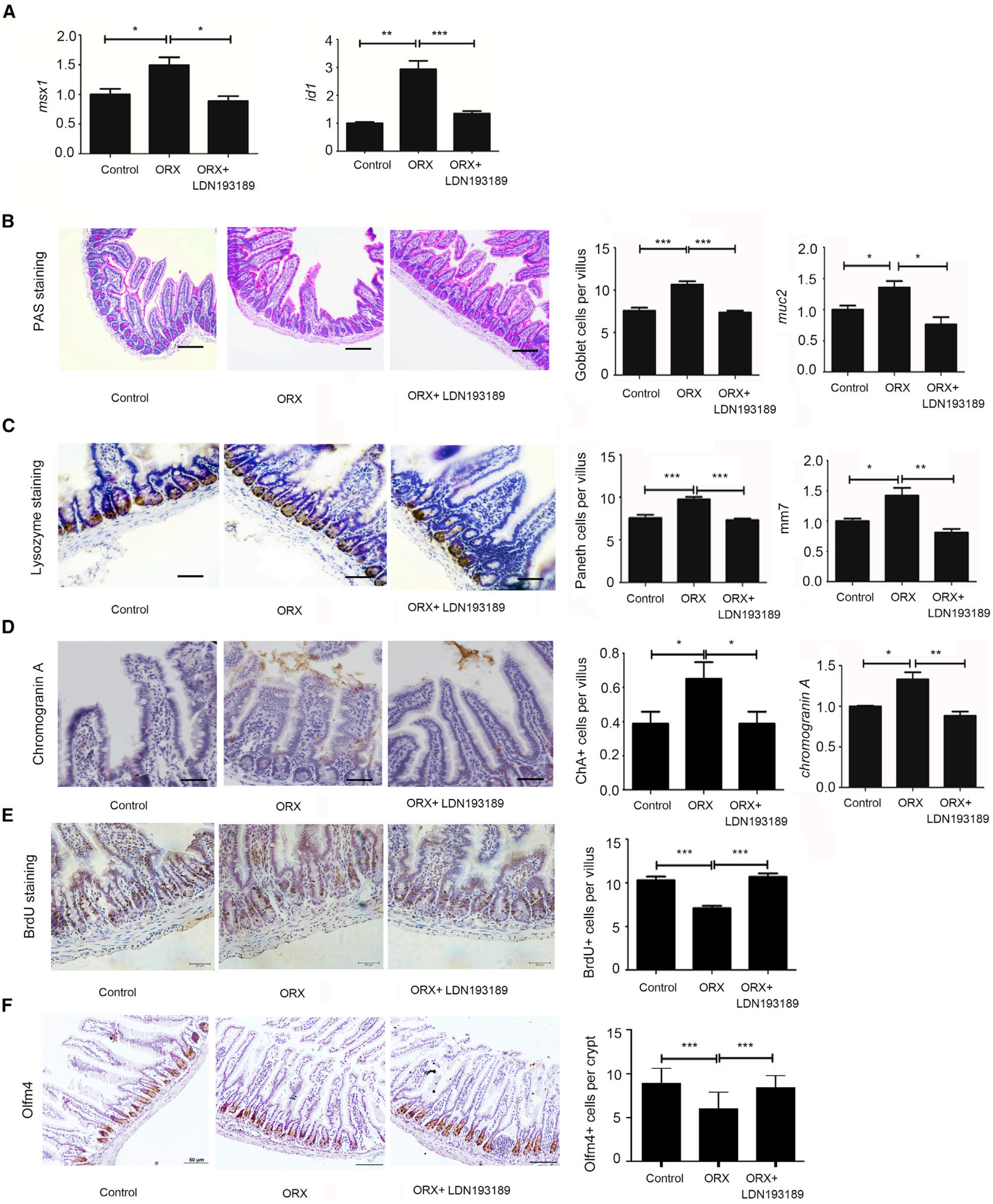

Figure 6. Androgen Played a Role Similar to Inhibitor of the BMP Pathway

A model of inhibition of the BMP pathway was constructed to elucidate the mechanism of androgen's effect on intestinal epithelial differentiation and proliferation. For counting of secretory and proliferative cells, 10 villi were randomly chosen for the goblet cells and

(legend continued on next page)

canonical Wnt pathway, which promoted proliferation through β-catenin-mediated signaling. In addition, the expression of a few Wnt ligands, such as Wnt 4, 5a, 5b, 2b, and R-spondins 1, 2, and 3, was not significantly changed. We observed that stroma cultured with a particular concentration of androgens expressed more BMP antagonists (*Chordin, Angptl2*) and reduced *Bmp4*. TGF-β1 and its downstream signals (*cdkn1a*, *cdkn2a*, and *cdkn2b*) were also downregulated by androgens. However, we were unable to confirm whether androgens directly inhibit the expression of *Bmp4*.

Next, we generated a model by administering LDN193189, a selective inhibitor of the BMP pathway, in ORX males. Outcomes were consistent with the reverse effect of exogenous androgens. BMP antagonists reversed the increase of secretory lineages and decrease of cellular proliferation caused by ORX. They also inhibited upregulation of BMP downstream signals (*Id1* and *Msx1*), further proving that androgens may serve as selective inhibitors of the BMP pathway.

In conclusion, the present study investigated the effect of androgens in promoting proliferation and inhibiting differentiation. According to our results, androgens bind to AR located in the mesenchyme and cause changes in the ISC niche mediated by the mesenchyme. The generation of inhibitors of canonical Wnt signaling was downregulated, leading to enhancement of Wnt/β-catenin signaling in ISCs. TGF-β1 and Bmp4 from mesenchyme were downregulated, and inhibitors of the BMP pathway were increased, causing reduced *Bmp4/Id1/Msx1* and *Tgfβ1/Cdkn1a/Cdkn2a/Cdkn2b* signaling in ISCs (Figure 7).

EXPERIMENTAL PROCEDURES

Experimental Animals

All experiments were performed under the guidance of the Medical Ethics Committee of the School of Basic Medical Sciences, Shandong University. C57BL/6J mice were bred at the Center of Experimental Animals, Shandong University.

ORX Model and Hormonal Manipulation

The ORX model was created in male mice that were 6–8 weeks of age (n = 6 for each group). All mice were routinely bred after arrival for 1 week to adapt to the new environment. Then, testicular castration was performed as previously described (Rombaut et al., 2016). Briefly, each mouse was anesthetized with 2% pentobarbital sodium and attached to the operation table, lying on its back. The scrotum, with the hair nearby cut off, was sterilized. The local skin, fascia, and peridydmis were cut layer by layer to create an incision, exposing the testis. For mice in the ORX and ORX+T groups, spermatic cords were ligated, and testes were cut off, while for mice in the control group, no disposal occurred and the wound was closed. DHT (MedChemExpress, New Jersey, 0.53 mg/kg/day) was injected intraperitoneally (i.p.) into ORX+T group mice from the first day after surgery. Meanwhile, ORX group and sham operation (group C) mice received DMSO solution (1%). Injections were arranged at the same time every day. Four weeks later, mice were sacrificed via cervical dislocation.

OVX Model and Hormonal Manipulation

The OVX animal model was created in mice that were 6–8 weeks of age (n = 6 for each group). All female mice had a prebreeding period for 1 week, followed by OVX operation (Amos-Landgraf et al., 2014). Every mouse was anesthetized with 2% pentobarbital sodium and attached to the operation table, in the prone position. The lower back, with the hair nearby cut off, was sterilized. The local skin and fascia were cut layer by layer to create an incision, exposing the ovary, which was isolated. OVX mice were randomly separated into two groups, OVX and OVX+T. DHT was injected i.p. into OVX+T group mice from the first day after surgery. Meanwhile, OVX group mice received DMSO solution (1%). Injections were arranged at the same time every day. Four weeks later, mice were sacrificed via cervical dislocation.

Inhibition of AR Model

2-Hydroxy flutamide is a competitive inhibitor of AR. The 2-hydroxy flutamide injection was composed of 2-hydroxy flutamide (10 mg/kg/day, Cayman Chemical, 15271) and normal saline (NS). Ten male mice were randomly separated into two groups, control group (0.1 mL NS) and flutamide group (0.1 mL 2-hydroxy flutamide injection). After 10 days of injections, all mice were sacrificed via cervical dislocation.

Inhibition of BMP Pathway Model

LDN193189 (APExBIO, A3545) is a selective BMP pathway inhibitor. Eighteen male mice were randomly separated into three groups designated as control, ORX, and ORX+LDN193189. Similar to the ORX model, mice in the control group received sham operation and those in groups ORX and ORX+LDN193189 underwent testicular castration. Mice in group ORX+LDN193189 were injected

enteroendocrine cells, and 10 crypts were randomly captured for the Paneth cells and $BrdU^+$ cells. Data, indicated as mean ± SD, correspond to three independent experiments (n = 6 mice/group/experiment).
(A) For analysis of *msx1* and *Id1*, downstream molecules of the BMP pathway, quantitative real-time PCR was performed.
(B) For the goblet cell count per villus, PAS staining was performed. Scale bar, 100 μm.
(C) For the Paneth cell count per crypt, IHC was performed with primary antibody to lysozyme. Scale bar, 20 μm.
(D) For the enteroendocrine cell count per villus, IHC was performed with the primary antibody to chromogranin A. Scale bar, 20 μm.
(E) For the $BrdU^+$ cells per crypt, IHC was performed with primary antibody to BrdU. Scale bar, 20 μm.
(F) For the $Olfm4^+$ cells per crypt, IHC was performed with primary antibody to Olfm4. Scale bar, 50 μm. *p < 0.05, **p < 0.01, and ***p < 0.001.

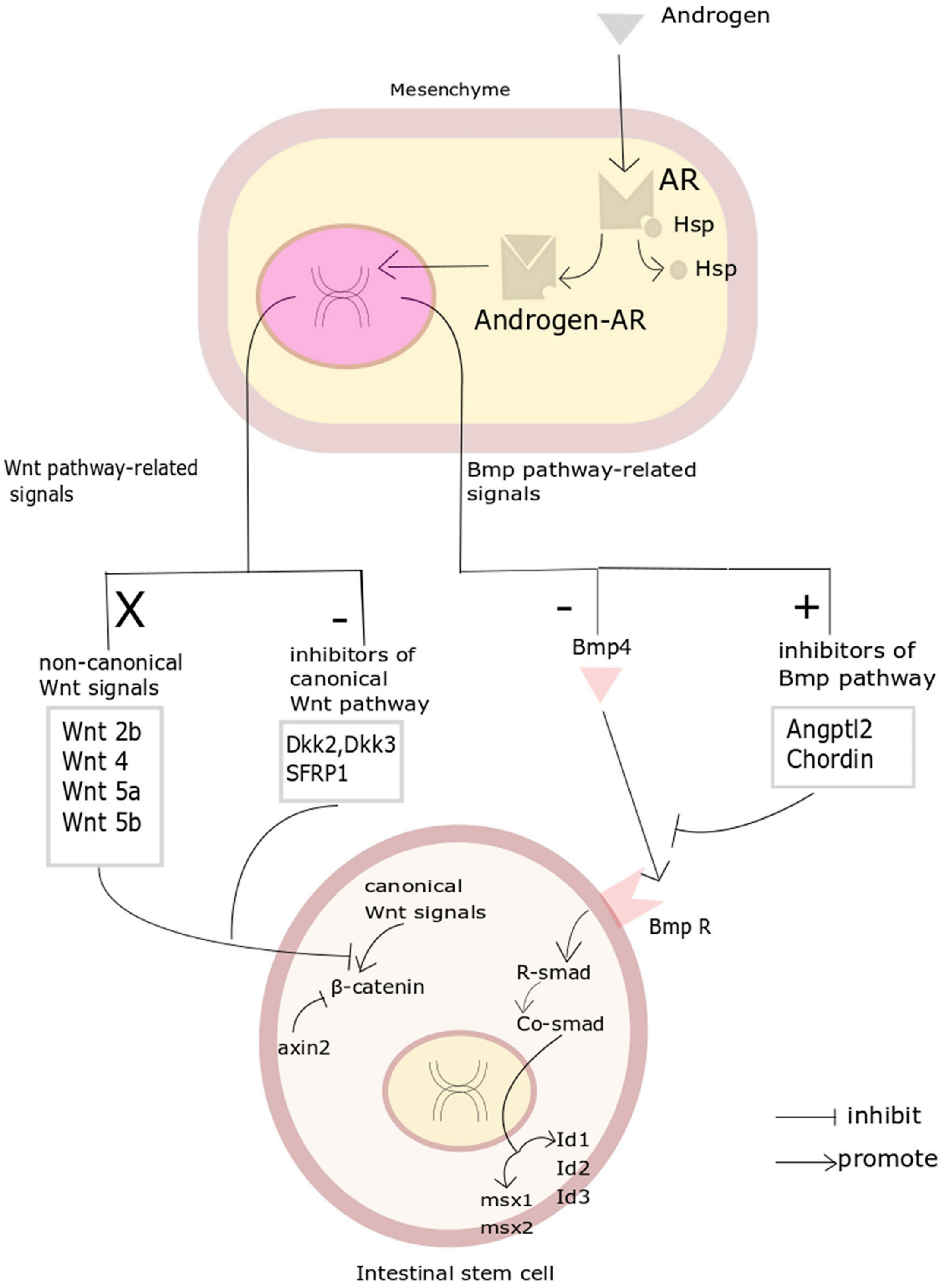

Figure 7. Androgen Might Regulate Proliferation and Differentiation in Mesenchyme-Mediated ISC Niche

Graphical view of androgen's effect on ISC renewal and the underlying mechanism. Wnt-related signals and Bmp-related signals mediated by mesenchyme are changed after activation of AR. AR, androgen receptor.

with LDN193189. Intraperitoneal administration was performed (3 mg/kg) for 5 consecutive days at intervals of 12 h (Bae et al., 2018). Control and ORX groups received DMSO solution (1%).

Tissue Processing

All animals were sacrificed by cervical dislocation. An i.p. injection of 0.1 mL BrdU (1%, Sigma, B5002) was administered 2 h before sacrifice. Tissue processing was performed with the cooperation of two researchers: one was responsible for animal sacrifice and one counted ileocecal junctions and placed the ileum and rectum into iced PBS. Contents in the intestinal tract were flushed carefully. The first 1 cm of ileum from the ileocecal junction was preserved in 1 mL RNA-fixer (Aidlab, lot 282133AX) at −80°C. The second 1 cm of ileum was preserved for western blot. Then another 5 cm of ileum was cut and incubated in 4°C paraformaldehyde solution overnight, and then transferred into 50%, 70%, 90%, and 100% ethanol and xylene successively for fixation. Fixed tissues were embedded into paraffin and made into 4 μm sections on glass slides.

Staining and Counting of Cells

After deparaffinization, IHC analyses were performed on glass sections. BrdU-positive (BrdU$^+$) cells were stained via the GTVision Detection System/Mo&Rb kit (GeneTech, lot GK600505) with BrdU (Bu20a) mouse mAb primary antibody (Cell Signaling, lot 5292S). Enteroendocrine cells were stained via Chromogranin A (AbCam, lot ab15160) antibody. Paneth cells were stained via the SPlink Detection Kit (biotin-streptavidin HRP detection systems) (ZSGB Bio, lot SP-9001) with polyclonal rabbit anti-human lysozyme primary antibody (Dako, lot 00074097). The staining of stem cells used anti-Olfm4 antibody (Cell Signaling, lot 39141S). For AR, an SP-9001 kit was used with Rb pAb (AbCam, lot ab74272). Specifically, we used PAS staining for goblet cells, which is performed using AB-PAS kits (Baso, lot BA-4121) and MST-8038 kit (MXB, lot 1811198038). And for enterocytes, the alkaline phosphatase staining of enterocytes was performed using the Vulcan Fast Red Chromogen kit 2 (Biocare Medical, FR805). All analyses followed standard protocols from the manufacturers. Cell counting was completed using an optical microscope (Nikon). For BrdU$^+$ cells and Paneth cells, 10 crypts were randomly captured in each mouse. For goblet cells and enteroendocrine cells, 10 villi were randomly chosen in each mouse. For enterocytes, 10 villi were randomly chosen in each mouse and cell counting was finished by ImageJ. Representative microscope images were captured by a digital camera mounted on the microscope.

RNA Isolation

RNA was extracted from intestines using the RNA kit (lot RN28) according to the manufacturer's instructions, which include the elimination of genomic DNA. Total RNA concentration and purity were measured using spectrophotometry (Thermo Fisher Scientific), and 1.0 μg of total RNA was used to perform cDNA synthesis.

cDNA Synthesis

First-strand cDNA was synthesized using a FastKing RT Kit (with gDNase) (Tiangen, lot KR116).

Quantitative Real-Time PCR

Quantitative real-time PCR was performed with the Sensifast Sybr No-Rox Kit (GC Biotech) with Hot-Start Taq polymerase. The temperature profile was as follows: (1) 95°C for 30 s; (2) 40 cycles of 95°C for 10 s, 56°C for 30 s, and 72°C for 30 s; and (3) 95°C for 15 s, 60°C for 60 s, and 95°C for 15 s. Quantitative real-time PCR was performed minimally in at least three independent biological replicates for each sample.

Primer Design

Necessary primers for quantitative real-time PCR analysis were designed using Primer-Blast. These primers were synthesized by the Beijing Genomics Institute. Primer sequences for analyzed genes are shown in Table S1.

Western Blotting

Fresh intestinal tissues were washed in 1 mL of ice-cold PBS several times. Tissue and frozen steel beads were placed into a 2 mL centrifuge tube, and tissues were homogenized and lysed in lysis buffer with a protease and phosphatase inhibitor cocktail (Sigma). After clearing by centrifugation, protein concentrations in the supernatant were determined using a spectrophotometer (Thermo Fisher). Proteins were separated by SDS-PAGE and transferred to polyvinylidene difluoride membranes (Immobilon-P, Millipore). Significant results are shown, and similar results were obtained in at least three independent experiments. The primary antibodies were anti-β-catenin (BD Biosciences, lot 610154), anti-active β-catenin (Cell Signaling, lot D13A1), anti-psmad1/5 (Cell Signaling, lot 41D10); and anti-math1 (Santa Cruz, lot sc-136173). Proteins were detected by ECL WB detection regent (Thermo Fisher).

Crypt Isolation and Culture

The entire small intestines were obtained from male wild-type C57BL mice. Separated intestines were dissected longitudinally and washed with ice-cold PBS. While the intestines were kept immersed in ice-cold PBS the whole time, the wastes and villi in the intestines were gently scraped with a clean coverslip to maintain the integrity of the crypts. Next the intestines were cut into small segments (approximately 2–3 mm) and transferred to a 50 mL centrifuge tube with 20 mL ice-cold PBS. Segments were washed in ice-cold PBS until no impurities remained in the supernatant and then incubated in ice-cold PBS containing 30 mL EDTA (2 mM) for 30 min with gentle shaking at a rate of 100 rpm. After digestion, the intestinal pellets were washed with PBS two or three times. The supernatant from the first few washes contained a large amount of villi, so it was discarded during washing. The segments were gently washed in fresh PBS to remove the remaining villi, and the process was repeated several times with ice-cold PBS/10% fetal bovine serum (FBS). The collected supernatant was then passed through a 70 mm cell strainer. We collected the filtered liquid in a 50 mL centrifuge tube, and the suspension was used for subsequent crypt isolation. Crypts were enriched by centrifugation at 300 × *g* for 3 min. The supernatant was carefully removed after centrifugation, and the cells were resuspended in 6 mL of incomplete medium (Advanced DMEM/F12, N2, B27, penicillin-streptomycin [PS], NAC, and Glutamax) and transferred into a 15 mL tube. Cells were then counted using a phase-contrast microscope.

After the crypts were centrifuged for 5 min at 200 × *g*, the supernatant was removed, and cells were resuspended in Matrigel that was thawed at 4°C and plated in 48 well plates (50 μL per well, approximately 2500 crypts). The plate was incubated at 37°C and 5% CO_2 for 18 min, followed by the addition of 250 μL complete medium to each well. Generally, the crypt medium was changed every 2–3 days, and crypts were passaged every 7 days.

Stromal Isolation and Coculture

After crypt separation from the intestinal segments, the segments were pipetted 50 times to remove the majority of the remaining epithelial cells. Samples were then washed once with PBS and serum-free DMEM containing 1% Glutamax and 1% PS (Kabiri et al., 2014). Thereafter, intestinal segments were digested for 3 h in 6 mL of serum-free DMEM containing 1% Glutamax, 1% PS and 2 mg/mL collagenase/dispase (Roche). The digestion solution was replaced with new digestion solution every hour. Intestinal segments were pipetted several times, and the digested solution was collected every hour. Then, the solution was supplemented with 5% FBS to inhibit proteolytic activity and cellular aggregation. Next, segments were isolated by energetic pipetting and filtered through a 70 μm cell strainer, centrifuged at 400 × *g* for 4 min, resuspended once in PBS, and counted. At that time, stromal cells were mixed with epithelial cells or cultured for 3–5 days in RPMI 1640 containing 10% FBS, 1% PS, and 1% Glutamax. To test the effect of DHT in intestinal epithelial cells, intestinal crypts (usually 2500 crypts) were mixed fresh or cultured with stroma (10,000–20,000 cells) in 15 mL centrifuge tubes and pelleted by centrifugation at 400 × *g* for 3 min. The supernatant was gently removed, and using 50 μL Matrigel per well, samples were gently resuspended and directly seeded into preheated 48 well plates. The 48 well plates were incubated at 37°C for 18 min to allow the Matrigel to form a gel, and then each well was supplemented with 200 μL crypt complete medium.

Statistical Analysis of Data

All cell count data are presented as the mean ± SEM. Significance levels were calculated using Student's t test. Experiments with multiple comparisons were analyzed by ANOVA.

SUPPLEMENTAL INFORMATION

Supplemental Information can be found online at https://doi.org/10.1016/j.stemcr.2020.08.001.

AUTHOR CONTRIBUTIONS

X.Y., S.L., and Y.X. performed research, analyzed the data, and helped draft the manuscript; Y.Z. and C.L. performed research and analyzed the data; H.L., Y.J., and W.M. performed research. D.C. and J.L. were responsible for the design of the study, interpretation of data, and writing of the manuscript.

ACKNOWLEDGMENTS

This work was supported by grants from the National Natural Science Foundation of China (31571183), China; Taishan Scholars Program, China; National Key Research and Development Program of China (2016YFC1302203), China and Shandong Key Research and Development Program (2017GSF218032), China.

Received: May 26, 2019
Revised: August 4, 2020
Accepted: August 5, 2020
Published: September 10, 2020

REFERENCES

Amos-Landgraf, J.M., Heijmans, J., Wielenga, M.C., Dunkin, E., Krentz, K.J., Clipson, L., Ederveen, A.G., Groothuis, P.G., Mosselman, S., Muncan, V., et al. (2014). Sex disparity in colonic adenomagenesis involves promotion by male hormones, not protection by female hormones. Proc. Natl. Acad. Sci. U S A *111*, 16514–16519.

Auclair, B.A., Benoit, Y.D., Rivard, N., Mishina, Y., and Perreault, N. (2007). Bone morphogenetic protein signaling is essential for terminal differentiation of the intestinal secretory cell lineage. Gastroenterology *133*, 887–896.

Bae, J.S., Jeon, Y., Kim, S.M., Jang, J.Y., Park, M.K., Kim, I.H., Hwang, D.S., Lim, D.S., and Lee, H. (2018). Depletion of MOB1A/B causes intestinal epithelial degeneration by suppressing Wnt activity and activating BMP/TGF-beta signaling. Cell Death Dis. *9*, 1083.

Barker, N. (2014). Adult intestinal stem cells: critical drivers of epithelial homeostasis and regeneration. Nat. Rev. Mol. Cell Biol. *15*, 19–33.

Clevers, H. (2013). The intestinal crypt, a prototype stem cell compartment. Cell *154*, 274–284.

Clocchiatti, A., Cora, E., Zhang, Y., and Dotto, G.P. (2016). Sexual dimorphism in cancer. Nat. Rev. Cancer *16*, 330–339.

Edgren, G., Liang, L., Adami, H.O., and Chang, E.T. (2012). Enigmatic sex disparities in cancer incidence. Eur. J. Epidemiol. *27*, 187–196.

Ferlitsch, M., Reinhart, K., Pramhas, S., Wiener, C., Gal, O., Bannert, C., Hassler, M., Kozbial, K., Dunkler, D., Trauner, M., and Weiss, W. (2011). Sex-specific prevalence of adenomas, advanced adenomas, and colorectal cancer in individuals undergoing screening colonoscopy. JAMA *306*, 1352–1358.

Ghaleb, A.M., McConnell, B.B., Kaestner, K.H., and Yang, V.W. (2011). Altered intestinal epithelial homeostasis in mice with intestine-specific deletion of the Kruppel-like factor 4 gene. Dev. Biol. *349*, 310–320.

Gregorieff, A., Pinto, D., Begthel, H., Destree, O., Kielman, M., and Clevers, H. (2005). Expression pattern of Wnt signaling components in the adult intestine. Gastroenterology *129*, 626–638.

Haber, A.L., Biton, M., Rogel, N., Herbst, R.H., Shekhar, K., Smillie, C., Burgin, G., Delorey, T.M., Howitt, M.R., Katz, Y., et al. (2017). A single-cell survey of the small intestinal epithelium. Nature *551*, 333–339.

He, X.C., Zhang, J., Tong, W.G., Tawfik, O., Ross, J., Scoville, D.H., Tian, Q., Zeng, X., He, X., Wiedemann, L.M., et al. (2004). BMP signaling inhibits intestinal stem cell self-renewal through suppression of Wnt-beta-catenin signaling. Nat. Genet. *36*, 1117–1121.

Horvay, K., and Abud, H.E. (2013). Regulation of intestinal stem cells by Wnt and Notch signalling. Adv. Exp. Med. Biol. *786*, 175–186.

Jenny, M., Uhl, C., Roche, C., Duluc, I., Guillermin, V., Guillemot, F., Jensen, J., Kedinger, M., and Gradwohl, G. (2002). Neurogenin3 is differentially required for endocrine cell fate specification in the intestinal and gastric epithelium. EMBO J. *21*, 6338–6347.

Kabiri, Z., Greicius, G., Madan, B., Biechele, S., Zhong, Z., Zaribafzadeh, H., Edison, Aliyev, J., Wu, Y., Bunte, R., et al. (2014). Stroma provides an intestinal stem cell niche in the absence of epithelial Wnts. Development *141*, 2206–2215.

Kyryachenko, S., Kyrylkova, K., Leid, M., and Kioussi, C. (2012). Immunohistochemistry and detection of proliferating cells by BrdU. Methods Mol. Biol. *887*, 33–39.

Lee, C. (1996). Role of androgen in prostate growth and regression: stromal-epithelial interaction. Prostate Suppl. *6*, 52–56.

Mori-Akiyama, Y., van den Born, M., van Es, J.H., Hamilton, S.R., Adams, H.P., Zhang, J., Clevers, H., and de Crombrugghe, B. (2007). SOX9 is required for the differentiation of paneth cells in the intestinal epithelium. Gastroenterology *133*, 539–546.

Porter, E.M., Bevins, C.L., Ghosh, D., and Ganz, T. (2002). The multifaceted Paneth cell. Cell. Mol. Life Sci. *59*, 156–170.

Powell, D.W., Pinchuk, I.V., Saada, J.I., Chen, X., and Mifflin, R.C. (2011). Mesenchymal cells of the intestinal lamina propria. Annu. Rev. Physiol. *73*, 213–237.

Qi, Z., Li, Y., Zhao, B., Xu, C., Liu, Y., Li, H., Zhang, B., Wang, X., Yang, X., Xie, W., et al. (2017). BMP restricts stemness of intestinal Lgr5(+) stem cells by directly suppressing their signature genes. Nat. Commun. *8*, 13824.

Qiu, T., Grizzle, W.E., Oelschlager, D.K., Shen, X., and Cao, X. (2007). Control of prostate cell growth: BMP antagonizes androgen mitogenic activity with incorporation of MAPK signals in Smad1. EMBO J. *26*, 346–357.

Rombaut, C., Faes, K., and Goossens, E. (2016). The effect of a unilateral orchiectomy before gonadotoxic treatment on the contralateral testis in adult and prepubertal rats. PLoS One *11*, e0164922.

Shoshkes-Carmel, M., Wang, Y.J., Wangensteen, K.J., Toth, B., Kondo, A., Massasa, E.E., Itzkovitz, S., and Kaestner, K.H. (2018). Subepithelial telocytes are an important source of Wnts that supports intestinal crypts. Nature *557*, 242–246.

Siegel, R.L., Miller, K.D., Fedewa, S.A., Ahnen, D.J., Meester, R.G.S., Barzi, A., and Jemal, A. (2017). Colorectal cancer statistics, 2017. CA Cancer J. Clin. *67*, 177–193.

Siegel, R.L., Miller, K.D., and Jemal, A. (2015). Cancer statistics, 2015. CA Cancer J. Clin. *65*, 5–29.

Sigal, M., Logan, C.Y., Kapalczynska, M., Mollenkopf, H.J., Berger, H., Wiedenmann, B., Nusse, R., Amieva, M.R., and Meyer, T.F. (2017). Stromal R-spondin orchestrates gastric epithelial stem cells and gland homeostasis. Nature *548*, 451–455.

Sneddon, J.B., Zhen, H.H., Montgomery, K., van de Rijn, M., Tward, A.D., West, R., Gladstone, H., Chang, H.Y., Morganroth, G.S., Oro, A.E., and Brown, P.O. (2006). Bone morphogenetic protein antagonist gremlin 1 is widely expressed by cancer-associated stromal cells and can promote tumor cell proliferation. Proc. Natl. Acad. Sci. U S A *103*, 14842–14847.

Tian, Y., Ma, X., Lv, C., Sheng, X., Li, X., Zhao, R., Song, Y., Andl, T., Plikus, M.V., Sun, J., et al. (2017). Stress responsive miR-31 is a major modulator of mouse intestinal stem cells during regeneration and tumorigenesis. Elife *6*, e29538.

Torre, L.A., Bray, F., Siegel, R.L., Ferlay, J., Lortet-Tieulent, J., and Jemal, A. (2015). Global cancer statistics, 2012. CA Cancer J. Clin. *65*, 87–108.

van der Flier, L.G., and Clevers, H. (2009). Stem cells, self-renewal, and differentiation in the intestinal epithelium. Annu. Rev. Physiol. *71*, 241–260.

van der Flier, L.G., Haegebarth, A., Stange, D.E., van de Wetering, M., and Clevers, H. (2009). OLFM4 is a robust marker for stem cells in human intestine and marks a subset of colorectal cancer cells. Gastroenterology *137*, 15–17.

Vanuytsel, T., Senger, S., Fasano, A., and Shea-Donohue, T. (2013). Major signaling pathways in intestinal stem cells. Biochim. Biophys. Acta *1830*, 2410–2426.

Wei, E.K., Giovannucci, E., Wu, K., Rosner, B., Fuchs, C.S., Willett, W.C., and Colditz, G.A. (2004). Comparison of risk factors for colon and rectal cancer. Int. J. Cancer *108*, 433–442.

Yan, K.S., Janda, C.Y., Chang, J., Zheng, G.X.Y., Larkin, K.A., Luca, V.C., Chia, L.A., Mah, A.T., Han, A., Terry, J.M., et al. (2017). Non-equivalence of Wnt and R-spondin ligands during Lgr5(+) intestinal stem-cell self-renewal. Nature *545*, 238–242.

Stem Cell Reports, Volume *15*

Supplemental Information

Androgen Maintains Intestinal Homeostasis by Inhibiting BMP Signaling via Intestinal Stromal Cells

Xin Yu, Shiguang Li, Yiming Xu, Yundi Zhang, Wenlong Ma, Changchun Liang, Haodong Lu, Yuge Ji, Chuanyong Liu, Dawei Chen, and Jingxin Li

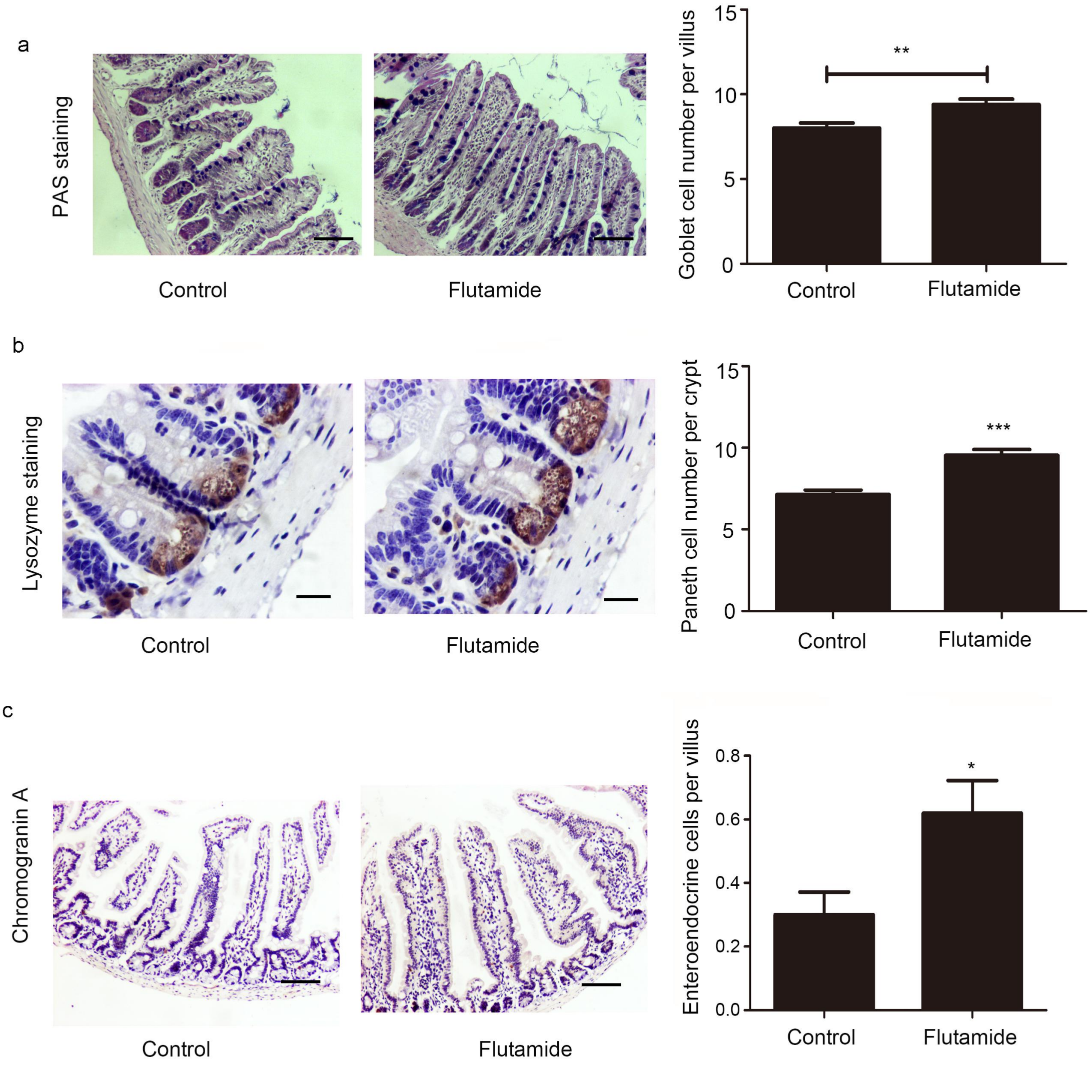

Fig.S1. Inhibition of AR model was constructed to analyze the change of three secretory lineages. Data, indicated as mean ± SD, correspond to three independent experiments (n=5 mice/group/experiment). (a) For the goblet cells count per villus in inhibition of AR model PAS staining was performed. Scale bar, 100μm. (b) For the Paneth cells count per crypt in inhibition of AR model, IHC was performed with primary antibody to lysozyme. Scale bar, 50μm, (c) For the enteroendocrine cells count per villus in the inhibition of AR model, IHC was performed with the primary antibody to chromogranin A. Scale bar, 100μm.

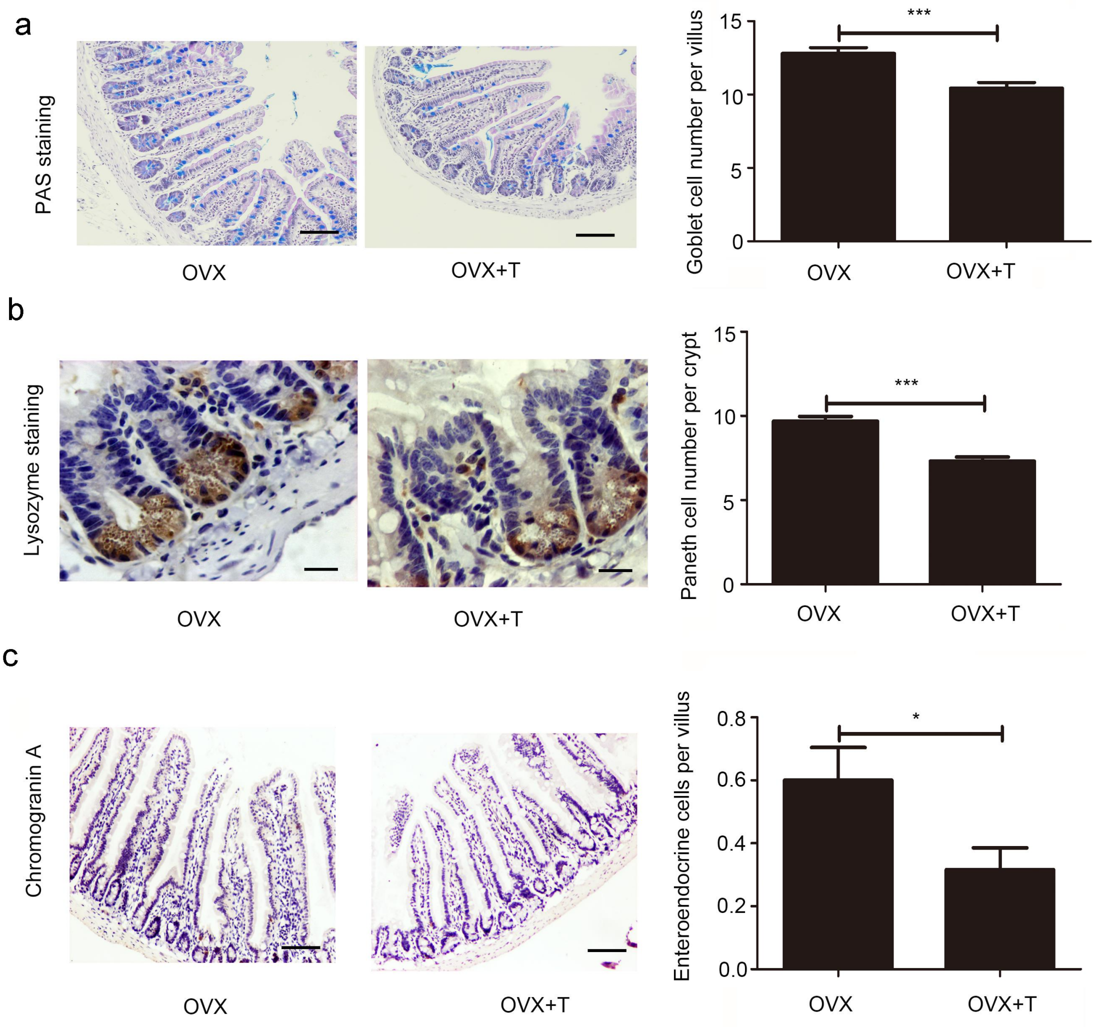

Fig.S2. OVX model was constructed to analyze the change of three secretory lineages. Data, indicated as mean ± SD, correspond to three independent experiments (n=6 mice/group/experiment). (a) For the goblet cells count per villus in OVX model, PAS staining was performed. Scale bar,100μm. (b) For the Paneth cells count per crypt in OVX model, IHC was performed with primary antibody to lysozyme. Scale bar, 50μm. (c) For the enteroendocrine cells count per villus in the OVX model, IHC was performed with the primary antibody to chromogranin A. Scale bar, 100μm.

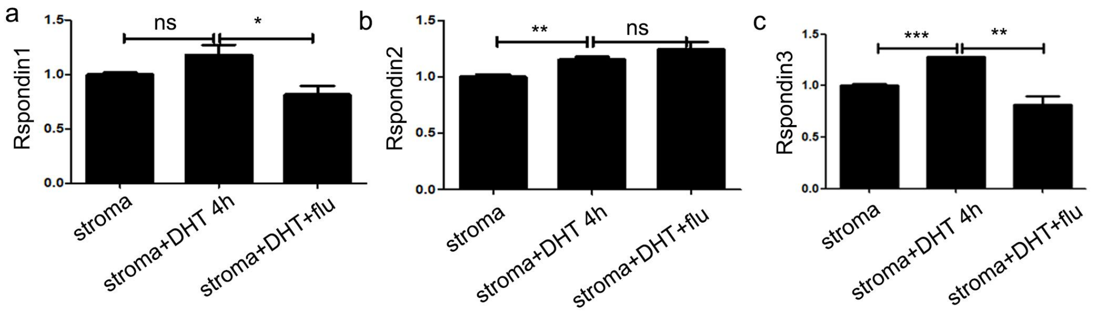

Fig.S3. Indicated mRNAs were analyzed by qRT-PCR. Data, indicated as mean ± SD, correspond to three independent experiments (n=4 mice/group/experiment). (a)*Rspo1,* (b)*Rspo2,* (c)*Rspo3.* ns means no significance. $^*P < 0.05$, $^{**}P < 0.01$, and $^{***}P < 0.001$

Table S1 Primer sequences of the analyzed genes

	forward	reverse
Ar	GGTCTTCTGGGGTGGAAAGT	GGGACCTTGGATGGAGAACT
Gadph	TGTGTCCGTCGTGGATCTGA	TTGCTGTTGAAGTCGCAGGAG
Chga	ATCCTCTCTATCCTGCGACAC	GGGCTCTGGTTCTCAAACACT
Angptl2	AAGCCATCAGGTAAGTCCTTTG	CCAGAGGATTGCCAGAGGATTA
Chrd	CCCATCCGTTCCGAGAAGG	ACTGAGGTTTGATGTTCTTGCAG
Sfrp1	CAACGTGGGCTACAAGAAGAT	GGCCAGTAGAAGCCGAAGAAC
Dkk2	CTGATGCGGGTCAAGGATTCA	CTCCCCTCCTAGAGAGGACTT
Dkk3	CTCGGGGGTATTTTGCTGTGT	TCCTCCTGAGGGTAGTTGAGA
Mmp7	CTGCCACTGTCCCAGGAAG	GGGAGAGTTTTCCAGTCATGG
Hes1	GATAGCTCCCGGCATTCCAAG	GCGCGGTATTTCCCCAACA
Wnt5a	CAACTGGCAGGACTTTCTCAA	CATCTCCGATGCCGGAACT
Wnt5b	CTGCTGACTGACGCCAACT	CCTGATACAACTGACACAGCTTT
Wnt4	AGACGTGCGAGAAACTCAAAG	GGAACTGGTATTGGCACTCCT
Wnt2b	CCGACGTGTCCCCATCTTC	GCCCCTATGTACCACCAGGA
Id3	CGACCGAGGAGCCTCTTAG	GGACGCGATAGGGAAGACC
Id2	GGTCTTCCTCCTACGAGCAG	CGATGGGCTTTAAGAGTGACAG
Msx2	TTCACCACATCCCAGCTTCTA	TTGCAGTCTTTTCGCCTTAGC
Cdkn1a	CCTGGTGATGTCCGACCTG	CCATGAGCGCATCGCAATC
Cdkn2a	CGCAGGTTCTTGGTCACTGT	TGTTCACGAAAGCCAGAGCG
Cdkn2b	CCCTGCCACCCTTACCAGA	CAGATACCTCGCAATGTCACG
Axin2	TGACTCTCCTTCCAGATCCCA	TGCCCACACTAGGCTGACA
Grem2	GGTAGCTGAAACACGGAAGAA	TCTTGCACCAGTCACTCTTGA
Nog	GCCAGCACTATCTACACATCC	GCGTCTCGTTCAGATCCTTCTC
Muc2	TTCGGCACGAGCAACTTTG	GGCAGGACACCTTGTCATTG
Bmp4	TTCCTGGTAACCGAATGCTGA	CCTGAATCTCGGCGACTTTTT

Tgfb1	CTCCCGTGGCTTCTAGTGC	GCCTTAGTTTGGACAGGATCTG
Klf4	CACCATGGACCCGGGCGTGGCTGCCAGAAA	TTAGGCTGTTCTTTTCCGGGGCCACGA
Elf3	TTCTGACTCCGGTGGAAGTGAT	CCCTTCTTATAGTCAGGAAAGCCA
Sox9	AAGTCGGTGAAGAACGGACAA	CAGCGCCTTGAAGATAGCATT
Ngn3	TCTCGCCTCTTCTGGCTTTC	CGGCAGATTTGAATGAGGGC
Atoh1	TATCCCGTCCTTCAACAACGA	TGGTCATTTTTGCAGGAAGCT
